# A simple and effective cryopreservation protocol for the industrially important and model organism, *Euglena gracilis*

**DOI:** 10.1016/j.xpro.2021.101043

**Published:** 2021-12-15

**Authors:** Mahfuzur R. Shah, Erin N. Morrison, Adam J. Noble, Scott C. Farrow

**Affiliations:** 1 Department of Cell Biology, Metabolism and Systems Biology, Noblegen Inc., 2140 East Bank Dr, Peterborough, ON, Canada; 2 Environmental and Life Sciences Graduate Program, Trent University, Peterborough, ON, Canada

**Keywords:** Cell Biology, Microbiology, Model Organisms, Biotechnology and bioengineering

## Abstract

*Euglena gracilis* is a source of high-value natural products. A major factor affecting consistent production of *Euglena* biomass is strain stability. Cryopreservation is a leading strategy for cell-line storage that helps ensure process reproducibility. We developed a simple cryopreservation protocol for heterotrophically cultured *Euglena* that enables the recovery of cells after 1 year with a cell viability of ≅80%. This protocol is suitable for labs interested in the long-term preservation of heterotrophic cultures of *Euglena* and related species.

## Highlights

• Cryopreservation protocol for heterotrophically cultivated *Euglena gracilis*
• Improved post-cryopreservation cell viability of ≅80%
• Protocol facilitates at least 1 year of cryopreservation
• Potentially applicable to the cryopreservation of other *Euglena* strains and species

## Graphical abstract

**Figure:**
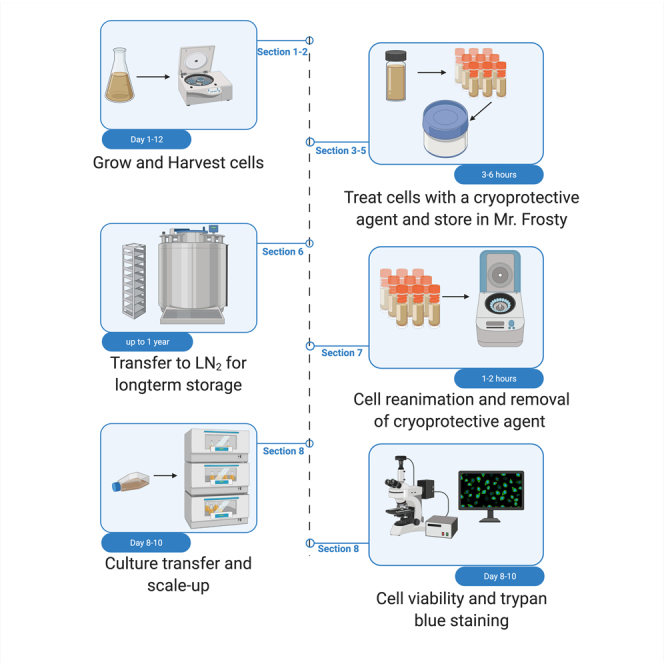

## Before you begin

### Development of the cryopreservation protocol

Consistent production of *E. gracilis* biomass is dependent on a preservation strategy that maintains the genetic fingerprint of the strain. Cryopreservation is a leading strategy for long-term cell-line storage that limits genetic drift, storage space, strain loss, cross-contamination, and permits facile clone transfer between facilities. Over the years, cryopreservation has been developed for a plethora of autotrophically grown microalgae and cyanobacteria, including Euglenophytes, by optimizing cryoprotectant solutions, freezing parameters, and recovery steps (Morris and Canning, 1978; Fleck et al., 2006; Day, 2007; Bui et al., 2013; Tessarolli et al., 2017; Kapoore et al., 2019). For example, Morris and Canning (1978) developed a method for *E. gracilis* but only achieved 30% cell recovery. We endeavored to improve upon such methods and create the first cryopreservation method for a commercial and heterotrophically grown strain of *E. gracilis*. By optimizing the cryoprotectant agent, freezing and thawing protocols and post recovery period, our cryoprotection protocol successfully preserves *Euglena gracilis* for at least 1 year with an enhanced cell recovery rate (≅ 80%) that permits faster process scaling.

### *E. gracilis* acquisition and media preparation

**Timing: Shipping + 1 day**

1. Obtain *Euglena gracilis* strain Z (UTEX 753; https://utex.org/products/utex-0753 Catalogue number: 0753).
2. Prepare glucose supplemented growth media (i.e., modified *E. gracilis* media – MEGM, https://www.ccap.ac.uk/wp-content/uploads/MR_EG.pdf).

### Preparation of cryopreservation equipment

**Timing: 1 day**

3. Fill the Mr. Frosty cooling unit with 250 mL isopropanol and equilibrate overnight at 4°C.
4. Pre-chill a cryo-box and cryo-rack (−196°C LN_2_) for long-term storage in LN_2_.
5. Fill and prepare LN_2_ storage dewar.

**CRITICAL:** Steps 3 and 4 should be done the day prior to cryopreservation.

## Key resources table

**Table:** 

REAGENT or RESOURCE	SOURCE	IDENTIFIER
**Chemicals, peptides, and recombinant proteins**		

Methanol	Fisher Scientific	CAT # 67-56-1
Sodium acetate trihydrate	Fisher Scientific	CAT # AC123240000
Lab-Lemco powder	Thermo Fisher Scientific	CAT # LP0029B
Tryptone	Thermo Fisher Scientific	CAT # 211701
Yeast extract	Thermo Fisher Scientific	CAT # 212750
Calcium Chloride	Fisher Scientific	CAT # AC219170000
Glucose	Thermo Fisher Scientific	CAT # 15023021
Trypan Blue (0.4%) solution	Thermo Fisher Scientific	CAT # 15250061
70% Ethanol	Fisher Scientific	CAT # BP82031GAL

**Experimental models: Organisms/strains**		

*Euglena gracilis* strain Z	UTEX	UTEX 753

**Other**		

Mr. Frosty Passive freezing unit	Nalgene Nunc International	CAT # 5100-0001
Syringe filters (0.2 μm)	Basix	CAT # 13100106
1 L Erlenmeyer flasks, vented cap, Polycarbonate	Corning	CAT # 431147
Sterile syringes (60 mL)	Air-Tite	CAT # MS60
Serological pipettes (10 mL)	FroggaBio	CAT # SP 10-200
Cryo-vials (pre sterilized plastic screw cap, 2 mL)	Corning	CAT # 430659
Glass Universal Vials (20 mL)	Thermo Fisher Scientific	CAT #139-20ACT
Countess II FL Automated cell counter	Thermo Fisher Scientific	CAT # AMQAF1000
T-25 flasks	Fisher Scientific	CAT # 12-565-348
YSI Biochemistry Analyzer 2950 D	YSI Life Science	CAT # 527690
Polycarbonate storage boxes	Nalgene	CAT # 5026-1010
Nalgene 0.2 μm Filter Unit	Nalgene	CAT # 566-0020
Locator JR Plus Rack and Box Cryo System Nitrogen Storage Dewar	Thermo Scientific	CAT # CY50985
Long forceps (19 cm)	Fisher Scientific	CAT # 50-822-717
Compound Microscope with Camera: EVOS FL AUTO AMAFD 1000	Life Technologies	SN # 1313-178C-098
Shaking Incubator with Temperature Control, ISF-4-V	Adolf Kohner AGI	SN # 88703-6
Heated water-bath, ISOTEMP 205	Fisher Scientific	CAT # 15-462-5
Benchtop Microcentrifuge, Sorvall Legend Micro 21	Thermo Scientific	CAT # 5002436
Benchtop Centrifuge, Sorvall ST 16	Thermo Scientific	CAT # 75004241
–80°C freezer	Forma Scientific	SN # 21094-2777
Spectrophotometer, SpectraMax - M3 Multi mode microplate reader	Molecular Devices	CAT # M3
PPE: lab coat, cryo-gloves, cryo-apron, protective goggles	N/A	N/A
Class II Biological Safety Cabinet, 1284 REL-3	Thermo Forma	SN # 45346

## Materials and equipment

**Table:** Glucose Supplemented Growth Media (i.e., MEGM)

Reagent	Final concentration	Amount
Sodium acetate trihydrate	1 g/L	1.0 g
Lab-Lemco powder	1 g/L	1.0 g
Tryptone	2 g/L	2.0 g
Yeast extract	2 g/L	2.0 g
Calcium chloride	1 g/L	1.0 g
Glucose	15 g/L	15.0 g

∗Add constituents above and make up to 1.0 L with deionized water. Growth medium should be sterilized by autoclaving at 15 psi, 121°C for 15 min or using a 0.2 μm filtration apparatus.

***Alternatives:*** We recommend MEGM but *E. gracilis* can also be grown in EG:JM media (https://www.ccap.ac.uk/wp-content/uploads/MR_EG_JM.pdf), Cramer-Myers media (Cramer and Myers, 1952), and Koren-Hutner media (Koren and Hutner, 1967).

## Step-by-step method details

### *E. gracilis* growth

**Timing: 10–12 days**

This section describes the growth and culturing of *E. gracilis* cells from stock in preparation for harvesting. Unless otherwise indicated all culturing, harvesting and subsequent sections should be carried out under sterile conditions.

1. Streak out fresh *E. gracilis s*train Z on glucose supplemented growth media + Agar (1.5%) using a sterile loop in a biosafety cabinet (or equivalent aseptic technique) and grow in the dark for 5–7 days (28°C) (Figures 1A–1C).

**Figure 1:**
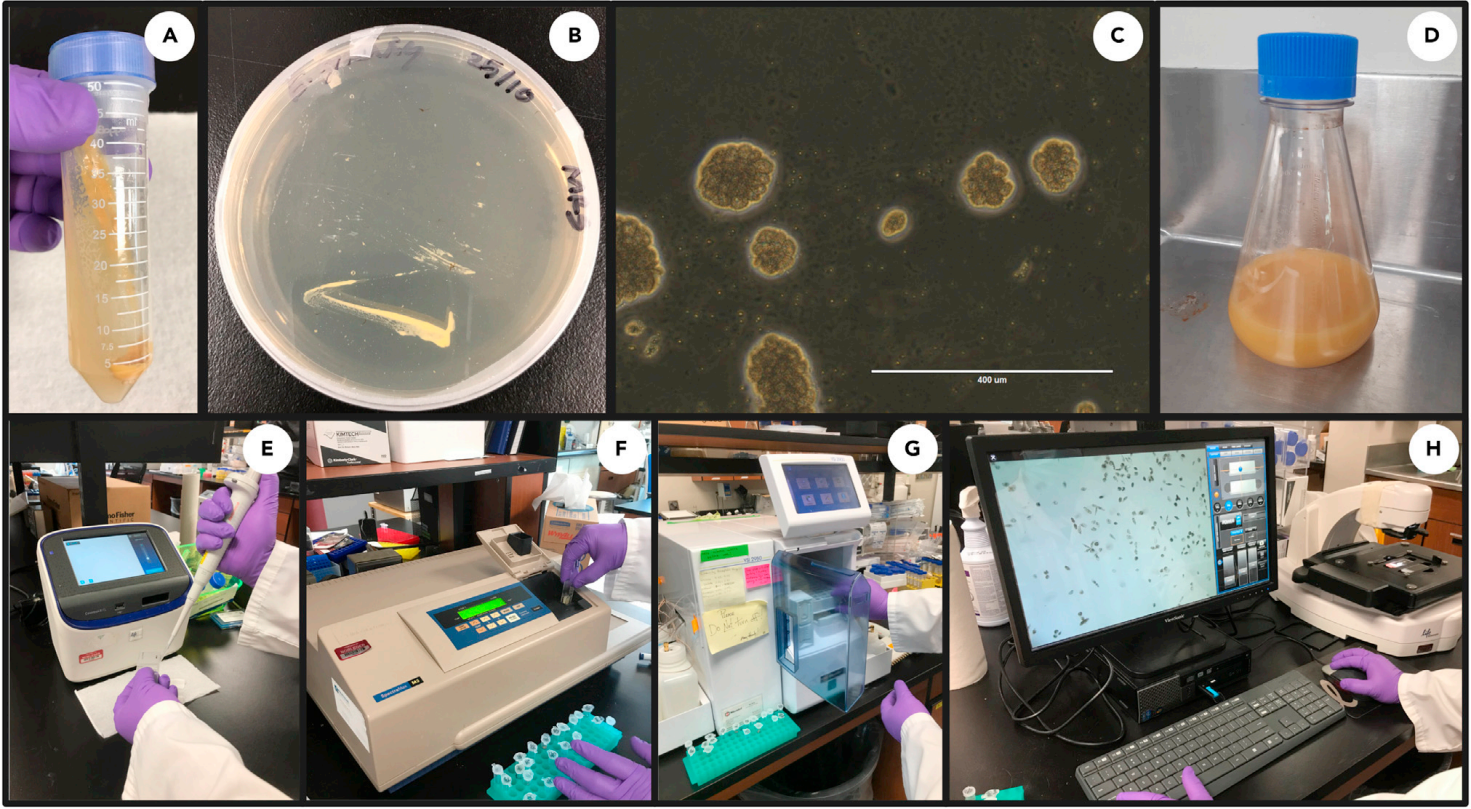
*E. gracilis* cell growth and monitoring (A and B) *E. gracilis* cells grown on an agar slant (A) or plate (B) take on a pale-yellow appearance. (C) Healthy colonies contain 10–90 round, pulsating cells (10× magnification, phase-contrast); (D) Cells grown in 200 mL glucose supplemented growth media; (E) Cell density determination (Countess II FL Automated cell counter); (F) OD measurement (Spectrophotometer); (G) Glucose measurement (YSI); (H) Morphology monitoring of *E. gracilis* cells.
2. Inoculate 200 mL of fresh glucose supplemented growth media (Figure 1D) with *E. gracilis* cells (≅3.5 × 10^6^ cells/mL) from step 1 using a sterile loop and grow heterotrophically in the dark (28°C, 120 rpm) until the glucose concentration is measured below 1 g/L (Figure 1G; ≅3 days). This equates to the late log or early stationary growth phase whereby cell count measures ≅ 12 × 10^6^ cells/mL (Figure 1E) or at OD_600_ measures ≅ 4.8 (Figure 1F).
  a. Growth of cultures can be monitored using a spectrophotometer and/or a cell counter, and glucose can be monitored using a YSI Biochemistry Analyzer 2950 D or equivalent technique (Figure 1). We recommend monitoring cell morphology using a compound microscope equipped with a camera (Figure 1H).

### Cell harvesting

**Timing: 1–2 h**

This section describes the harvesting of cells for cryopreservation.

3. Transfer 10 mL of the 200 mL culture to a 15 mL conical tube in a biosafety cabinet (or equivalent aseptic technique) using a 10 mL serological pipette.

**CRITICAL:** 1 mL of sample should be used to assess cell viability. See: post-cryopreservation culturing and cell viability assaysstep 21.

4. Pellet cells by centrifugation at 500 *g* for 5 min at 19°C–22°C (Figure 2A).

**Figure 2:**
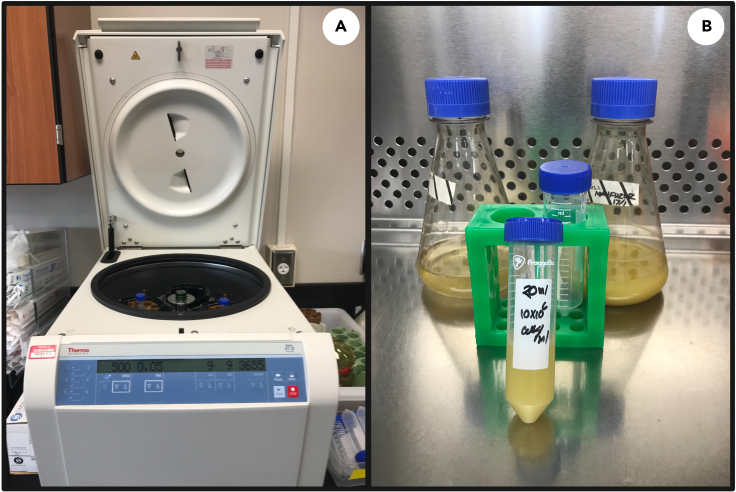
*E. gracilis* cell harvesting and incubation. (A) Cell harvesting by centrifugation; (B) Cell incubation for cell recovery.
5. After centrifugation, discard the supernatant and resuspend cells by gently pipetting in fresh, sterile glucose supplemented growth media to a final cell concentration of ≅ 10 × 10^6^ cells/mL.
6. Transfer the cell suspension to a sterile conical tube and let rest undisturbed at 19°C–22°C for a 30 min recovery period (Figure 2B).

**CRITICAL:** 30 min cell recovery at 19°C–22°C is critical after centrifugation.

7. Perform cryopreservation treatment according to the CPA treatment below.

### Preparation of the cryoprotective agent (CPA)

**Timing: 15 min**

These steps describe the preparation of the cryoprotectant agent under sterile conditions.

**Figure 3:**
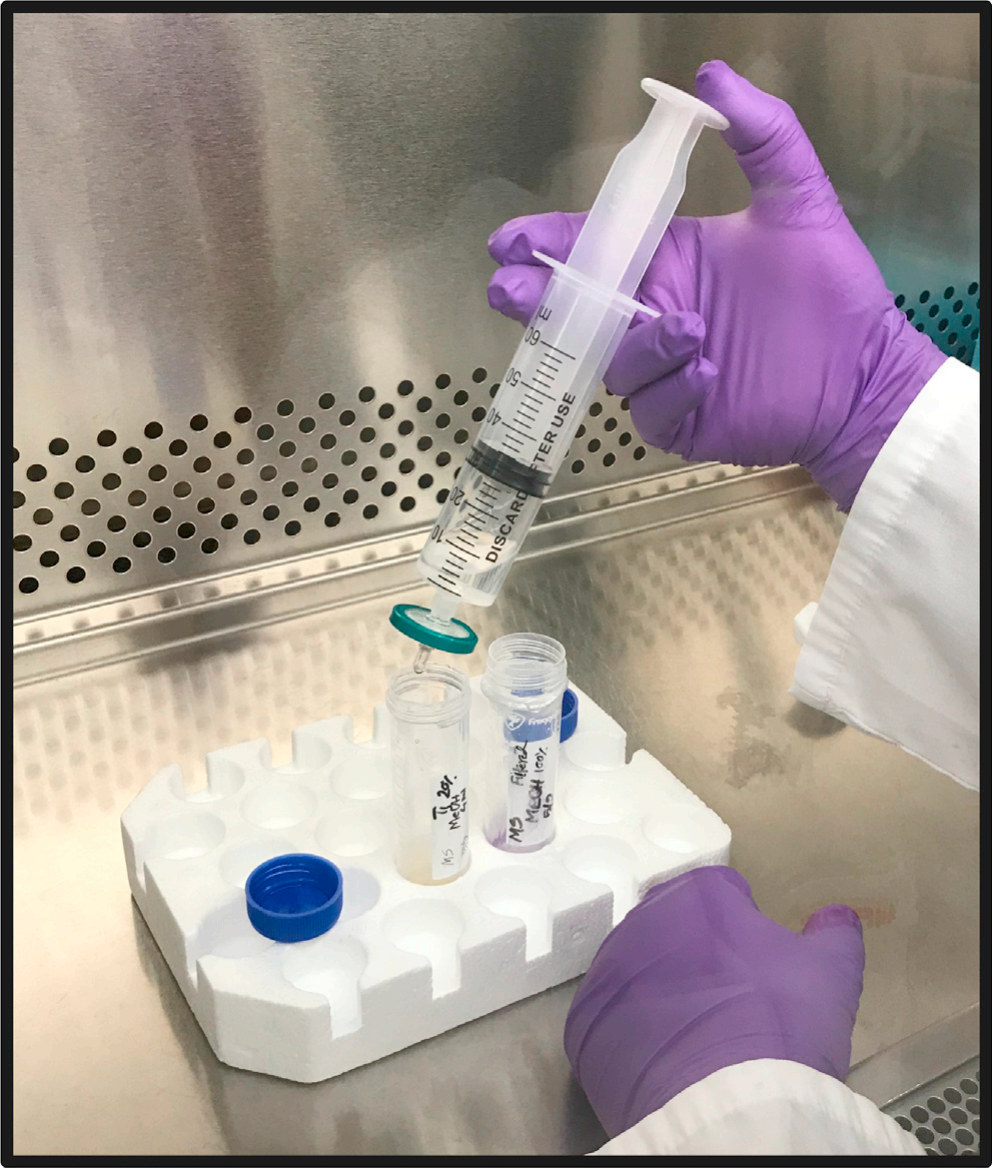
Preparation of CPA solution Filter sterilization of CPA including glucose supplemented growth media.

8. Add 2 mL of reagent grade methanol (MeOH) to 8 mL of sterile glucose supplemented growth media to make 10 mL of 20% (v/v) cryoprotective agent (CPA).
9. Filter-sterilize CPA into a 50 mL sterile conical tube using a 0.2 μm filter equipped with a 50 mL syringe (Figure 3) and keep at 19°C–22°C until use.

**CRITICAL:** CPA should be prepared fresh on the day of use. If CPA is stored for long periods or exposed to strong light, it can lose efficiency.

### Treatment of cells with CPA

**Timing: 30 min**

These steps describe the treatment of cells with CPA.

**Figure 4:**
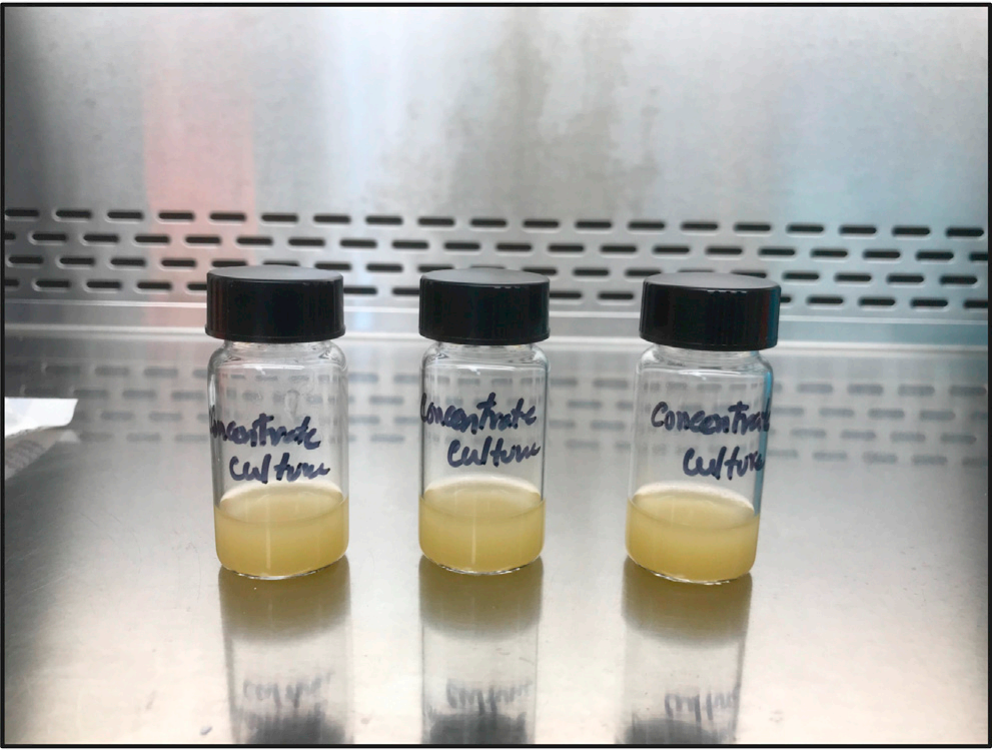
Treatment of *E. gracilis* cells with CPA (MeOH) solution
11. Seal vial(s) with parafilm and mix by gently inverting twice.

***Note:*** You can customize the volume at step 10 if the ratio remains 1-CPA:1-cells (v/v). We make extra CPA in case of spillage or when additional aliquots are desired.

10. Transfer 10 mL of *E. gracilis* cells (≅ 10 × 10^6^ cells/mL) from step 6 into a universal glass vial and add 10 mL of CPA to make a final 20 mL solution containing 10% MeOH (v/v) (Figure 4).

### Transferring of cultures to cryovial and equilibration

**Timing: 30 min**

This section describes the aliquoting of CPA treated cells.

**Figure 5:**
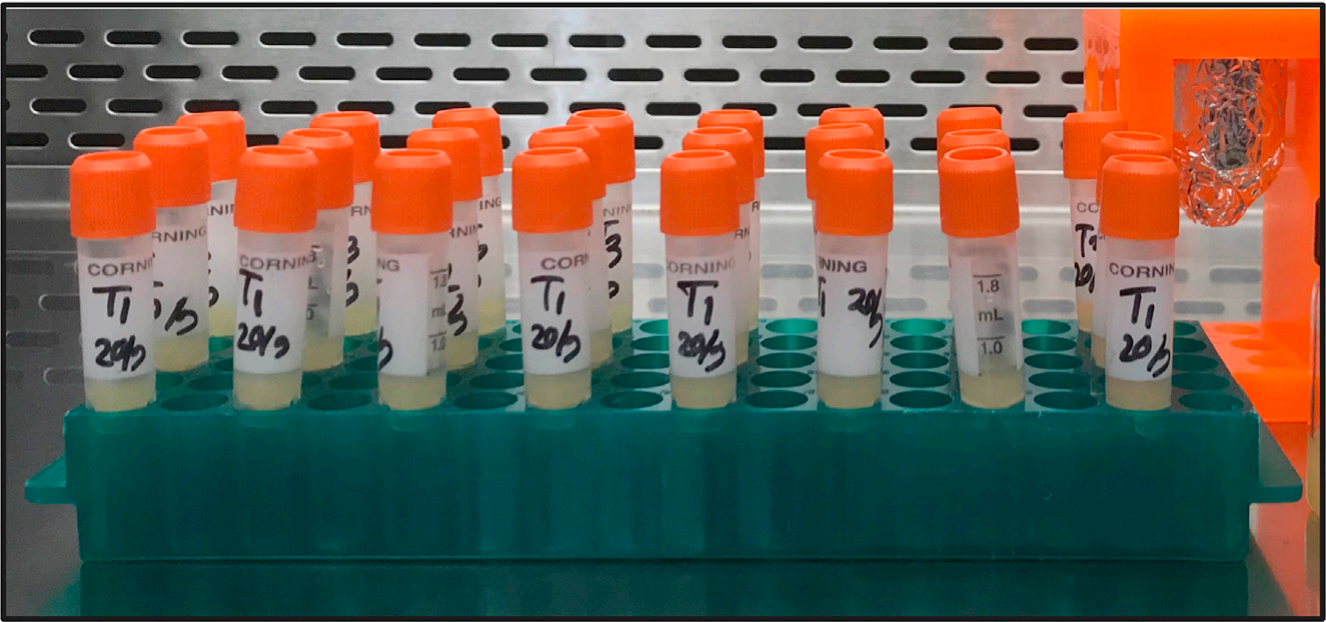
*E. gracilis* cells transferred to cryo-vials and equilibration

12. Aliquot 0.5 mL of CPA treated *E. gracilis* cells into sterile, 2.0 mL plastic screw cap vials.
  a. Repeat for a total of 18 vials - the capacity of the Mr. Frosty cooling container.
13. Seal cryo-vials with parafilm and incubate for 15 min at 19°C–22°C (Figure 5).

### Cooling and cryopreservation of *E. gracilis* cells

**Timing: 2 h**

This section describes the transfer of CPA treated cells, the preparation protocol for use of the Mr. Frosty and long-term storage of *Euglena* cells.

***Note:*** For cooling and cryopreservation of cells it is recommended to use the Mr. Frosty cooling device or a similar device like the Cool Cell™ which facilitate gradual cooling of cells. We tested an alternative cooling protocol by placing CPA treated cells directly at −80°C followed by LN_2_ but cells did not survive.

**Figure 6:**
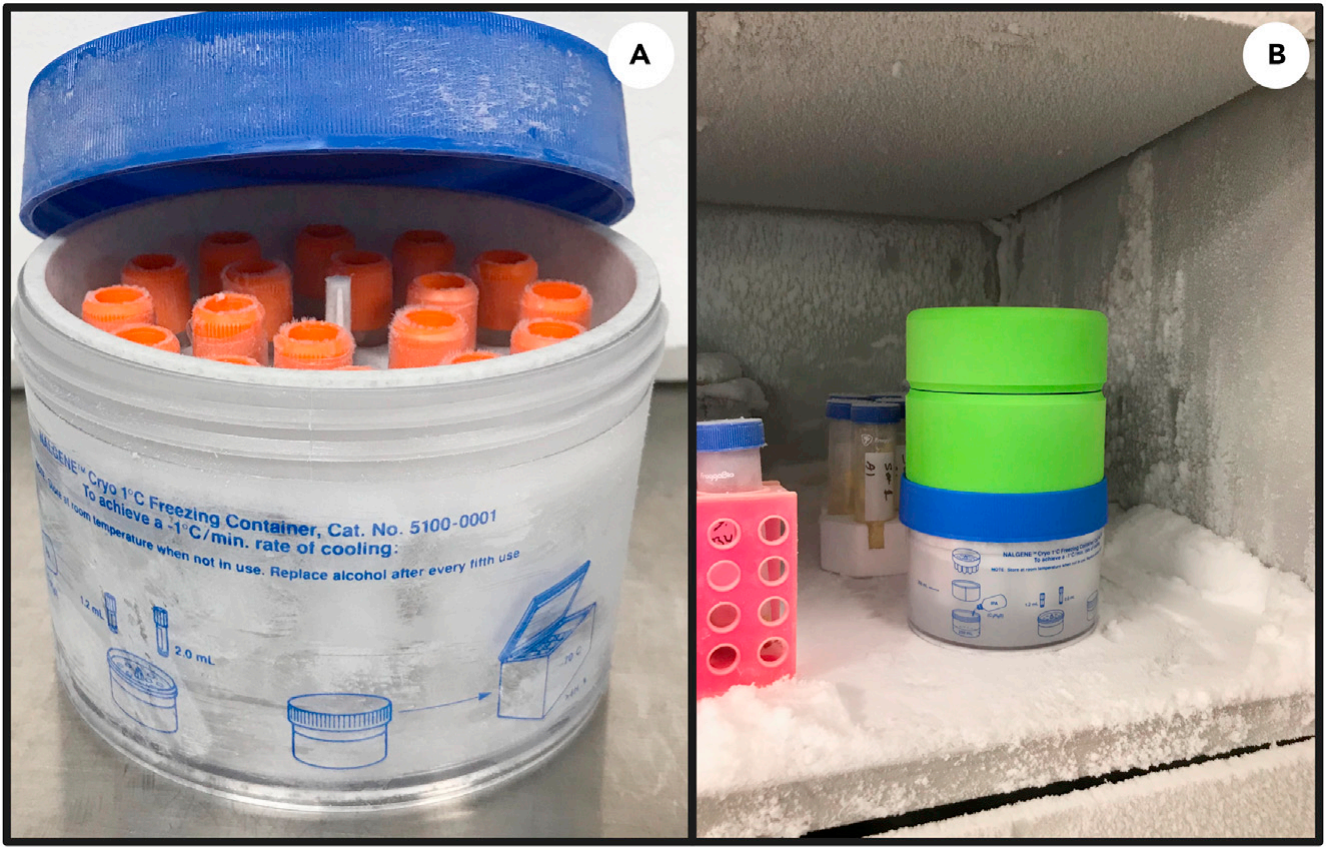
Cooling of *E. gracilis* cells in Mr. Frosty placed in a −80°C freezer (A) Cryovials organized in Mr. Frosty; (B) Mr. Frosty inside a −80°C freezer.

**Figure 7:**
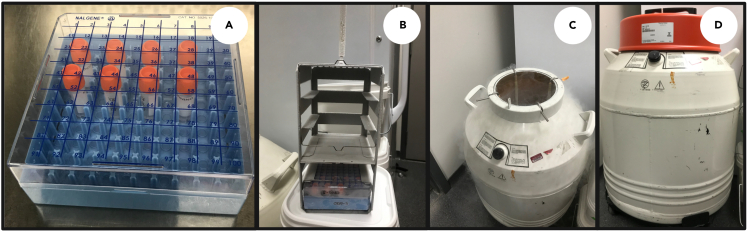
Placement of cryo-vials in LN_2_ filled dewar (A) Transferred cryo-vials from Mr. Frosty; (B) Cryo-box transfer to cryo-rack; (C) Cryo-rack to LN_2_ filled dewar; (D) *E. gracilis* cells cryopreserved in LN_2_ filled dewar.

14. Cooling Phase
  a. After incubation (step 13), immediately transfer cryo-vials containing CPA treated *E. gracilis* cells to the Mr. Frosty passive freezing system.
    **CRITICAL:** Step 14 should only proceed after the Mr. Frosty unit has been pre-treated with 250 mL of isopropanol and undergone overnight equilibration at 4°C. See: Before You Begin, step 3.
  b. Place the Mr. Frosty unit at -80°C and allow cells to rest for 1.5 h.
    i. The cooling rate during this step equates to −1 °C/min, and the temperature of the vial contents after 1.5 h is below −50°C (Figure 6).

15. Cryopreservation phase
  a. Take the pre-chilled cryopreservation vials containing the pre-chilled *E. gracilis* cells and quickly transfer them from the Mr. Frosty cooling unit into the pre-chilled (−196°C) cryo-box and cryo-rack for long term storage in LN_2_ (Figure 7).
  b. Monitor LN_2_ levels bi-weekly to ensure samples remain submerged in LN_2_.
    **Pause point:** Store cryopreserved cells for the desired time ensuring LN_2_ levels are maintained.
    **CRITICAL:** Ensure samples remain submerged in LN_2_ for the duration of storage.

### Thawing and recovery of *E. gracilis* cells after 1 year of cryopreservation

**Timing: 1–2 h**

This section outlines the steps required for preparation of cryopreserved cells for reanimation.

16. After the cryopreservation period (i.e., 1 year), remove the cryo-rack from LN_2_ and transfer the cryo-vials directly to a pre-warmed water bath (35°C, 1–2 min).

**Figure 8:**
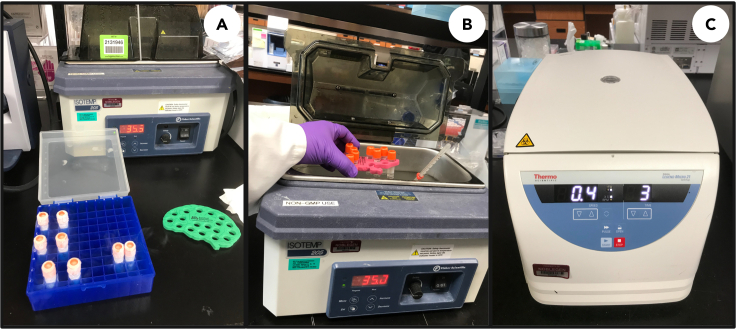
Thawing and recovery of *E. gracilis* cells after Cryopreservation (A) Cryo-vials transferred from LN_2_ filled dewar for thawing; (B) Thawing of cells in water bath; (C) Centrifugation and washing of cells to remove CPA.
18. To increase osmolarity and the total volume to 1.0 mL, carefully add 0.5 mL of fresh glucose supplemented growth media to the cell suspension and mix by gently pipetting.
19. Immediately pellet cells by gentle centrifugation (400 *g* for 3 min at 19°C–22°C) and discard the supernatant. Resuspend the pellet in 1 mL of fresh glucose supplemented growth media by gently pipetting, and incubate for 10 min at 19°C–22°C.
  a. Repeat step 19 once to remove remaining CPA.

***Note:*** We have successfully recovered cells after 1 week, 3 months, 6 months and 1 year of cryopreservation.

**CRITICAL:** Immerse cryovials to cover vial contents. This step should be done carefully to avoid agitation, and vial contents should be left immersed until cells are completely thawed (1–2 min) (Figure 8). The optimal temperature for thawing is 35°C (check with an analog thermometer).

17. After thawing, immediately remove cryo-vials from the water bath and sterilize the outer surface with 70% (v/v) ethanol. Rapidly transfer vials to a class II biosafety cabinet.

### Post-cryopreservation culturing and cell viability assays

**Timing: 8–10 days**

This section outlines the cultivation and assessment of cell viability for cells that have been reanimated following cryopreservation and storage. As in previous sections steps should be carried out under sterile conditions.

***Note:*** For culture vessels we recommend using T-25 flasks for post-cryopreservation culturing (step 20). T-25 flasks are advantageous as they can be placed directly under a microscope allowing for routine cell observation. Alternatively, one can use small, vented conical flasks but their use risks contamination as observing cells under the microscope requires opening the vessel to remove samples.

**Figure 9:**
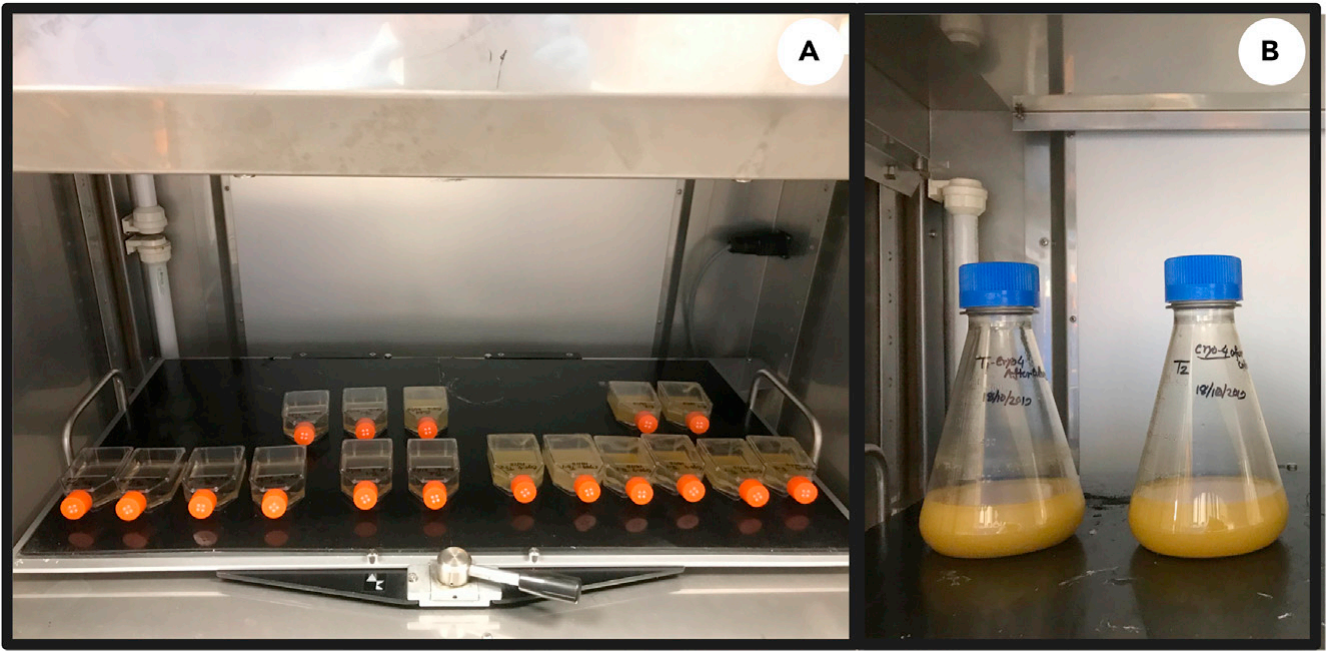
Post-cryopreservation culture and scale-up of *E. gracilis* culture (A) *E. gracilis* cells transferred to T-25 flasks and grown inside a shaker incubator; (B) Culture scale-up in 200 mL for further use.

**Figure 10:**
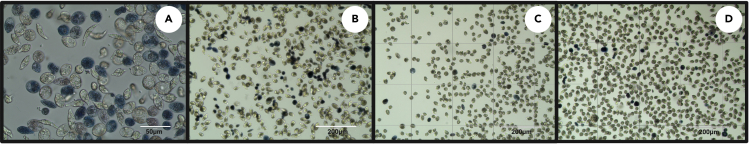
Cell viability assay using Trypan Blue (A) Before Cryopreservation (60×; 50 μm scale bar); (B) Before Cryopreservation (20×; 200 μm scale bar); (C) Immediately after thawing (20×; 200 μm scale bar); (D) Post cryopreserved and recovered culture (20×; 200 μm scale bar). Dark-blue cells are considered dead/inactive cells; Non stained cells are considered living/active cells.

20. Post-cryopreservation culturing
  a. Transfer 1 mL of washed *E. gracilis* cells to a culture vessel (T-25 flasks) containing 19 mL of fresh glucose supplemented growth media and grow cells on a rotary shaker at 80 rpm for 72–96 h at 28°C, under dark, heterotrophic conditions (Figure 9).
    **CRITICAL:** Take 1 mL of sample to assess cell viability. See: post-cryopreservation culturing and cell viability assays, step 21.
  b. After 72–96 h of growth (cell count ≅ 4.5 × 10^6^ cells/mL), transfer 20 mL of culture to 200 mL of fresh glucose supplemented growth media in a 1 L conical flask. Continue to grow culture under the same conditions but with a modified rotation speed, which should be changed to 120 rpm (Figure 9).
    i. Monitor cell density (cells/mL) and optical density (OD_600_) during post-cryopreservation culturing. Make morphological observations using a microscope to determine the health and viability of the culture (Figure 1).
21. Cell Viability Assays
  ***Note:*** To evaluate cryopreservation success, if resources permit, we recommend testing the success of the cryopreservation protocol at different time intervals (i.e., bi-weekly, monthly, quarterly, yearly) using the indicated cell viability assay.
  a. The viability of *E. gracilis* cells should be assessed using Trypan Blue (TB) and microscopy. This is done three times: (1) Before cryopreservation (step 3 of cell harvesting), (2) immediately after thawing (step 20a) and (3) immediately after the post-cryopreservation culturing period (step 20b).
    i. TB stains dead *E. gracilis* cells, which acquire a dark blue appearance. Living (viable) cells maintain their natural color. The following outlines the TB staining procedure (Figure 10).
      ***Note:*** When working with Trypan Blue follow all safety protocols as indicated by the manufacturer, wear appropriate PPE and discard waste in appropriate vessels.
  b. Add 1 mL of *E. gracilis* cell culture to a 2 mL tube and centrifuge at 2400 *g* for 5 min at 19°C–22°C and discard the supernatant.
  c. Add 1 mL of 0.4% TB solution to the cell pellet, mix by gently pipetting, and incubate at 19°C–22°C for 10 min.
  d. After incubation, centrifuge the sample at 2400 *g* for 5 min at 19°C–22°C, remove 900 μL of supernatant and replace with 900 μL of distilled water.
  e. Mix sample gently by pipetting and centrifuge at 2400 *g* for 5 min at 19°C–22°C to remove supernatant.
    i. Resuspend cells in 1 mL of distilled water and repeat the washing step three times.
  f. A minimum of 50 cells should be counted under the microscope (or microscope photo) to calculate viable and dead *E. gracilis* cells (Figure 1).
  g. Cell viability (%) is calculated using the following formula: (number of living cells/ number of total cells) × 100%. To avoid underestimating cell viability due to the presence of dead cells in the original cultures (step 3, cell harvesting), the viability of initial cultures should be calculated for comparison.

## Expected outcomes

The developed cryopreservation protocol is a simple and straightforward protocol for the long-term maintenance, storage, and recovery of heterotrophically grown *E. gracilis* strain Z. This protocol allows for long-term genetic stability and a storage strategy for companies and academic labs interested in *E. gracilis*. Additionally, it allows for easy strain recovery, transfer between locations and provides a useful starting point for developing subsequent methods for the maintenance and storage of other *Euglena* strains and species.

## Limitations

This protocol is dependent on the health of the starting cell culture, and adherence to the steps outlined above. This method has been optimized for heterotrophically cultured *Euglena gracilis* strain *Z* but serves as a starting point for *Euglena* grown using alternative carbon sources, and other *Euglena* strains and species.

## Troubleshooting

### Problem 1

Growth kinetics are slowed or there is a reduced culture growth rate (step 1).

### Potential solution

*E. gracilis* can grow over a wide temperature range (22°C–30°C) but we recommend growing *E. gracilis* cells at the experimentally optimized growth temperature (28°C). If slower growth rates are observed, it is recommended to check that all growing conditions are consistent with our suggested parameters. Sub-optimal growth can also be a consequence of an unhealthy culture, and therefore its health should be assessed before proceeding with cryopreservation. Healthy *E. gracilis cells* in liquid culture are elongated and actively moving. If they are rounded or non-motile, cultures should not be used for cryopreservation.

### Problem 2

Cryopreserved cells did not successfully reanimate indicating unsuccessful cryopreservation (step 8).

### Potential solution

CPA is a critical solution and should be prepared before harvesting cells for cryopreservation. Cryopreservation efficiency drops with CPA that has been stored for extended time frames or when it is exposed to light. It is not recommended to store CPA at 4°C or −20°C as this can also reduce efficiency. In addition, CPA must be mixed with growth media (1v:1v). It cannot be used with water. We have tested alternative CPA solutions (e.g., glycerol, DMSO) and concentrations and found that the final concentration of 10% MeOH is optimal for heterotrophically grown *E. gracilis.*

### Problem 3

Unsuccessful cell recovery (step 16).

### Potential solution

Unlike common laboratory organisms (i.e., yeast), *E. gracilis* does not have a cell wall which makes it sensitive to abrupt temperature changes. As such, the temperature and timing parameters described in this protocol must be strictly adhered to. For example, transfer of cells from LN_2_ to the water bath should be done quickly, as extended times at RT will impact recovery. We also tested different recovery temperatures (i.e., 28°C, 35°C and 37°C) and intervals (1, 2, and 3 min) but 35°C and 1–2 min was optimal. If viability remains poor, one must ensure that cells are healthy before harvesting, and that cells are not damaged during resuspension. Again, we recommend using the cell viability assay at the indicated steps before cryopreservation to assist with the evaluation of cell health.

### Problem 4

Contaminated cultures (step 2).

### Potential solution

All transferring steps must be carried out aseptically in a class II biosafety cabinet. While a biosafety cabinet is recommended, a laminar flow-hood and/or flame can be used to maintain dead airspace. All equipment, tubes etc. should be sterile prior to use and good aseptic technique should be used throughout.

## Resource availability

### Lead contact

Further information and requests for resources and reagents should be directed to and will be fulfilled by the lead contact, Scott Farrow (scott.c.farrow@gmail.com).

### Materials availability

This study did not generate new unique reagents.

## Data Availability

The published article includes all steps and indications for successful cryopreservation. Relevant cell count, viability information, and accompanying images are provided as a guide.
